# Hatchery-reared enhancement program for silver carp (*Hypophthalmichthys molitrix*) in the middle Yangtze River: monitoring the effectiveness based on parentage analysis

**DOI:** 10.7717/peerj.6836

**Published:** 2019-05-06

**Authors:** Huijuan Chen, Dengqiang Wang, Xinbin Duan, Shaoping Liu, Daqing Chen, Yun Li

**Affiliations:** 1 College of Animal Science and Technology, Southwest University, Chongqing, China; 2 Yangtze River Fisheries Research Institute, Chinese Academy of Fishery Sciences, Wuhan, China

**Keywords:** Yangtze River, Silver carp, Genetic characteristic, Effectiveness, Stock enhancement, Hatchery-reared

## Abstract

**Introduction:**

A hatchery-reared silver carp (*Hypophthalmichthys molitrix*) program has been intensively carried out since 2010 to enhance the rapidly declining fisheries production in the middle Yangtze River. However, only a little information regarding the effectiveness of the enhancement program has been reported. In this context, this study investigates on an enhancement program through monitoring the efficacy based on parentage analysis.

**Methods:**

A total of 1,529 hatchery-reared fish and 869 larvae were sampled from the middle Yangtze River in 2016 and 2017 and were genotyped by thirteen microsatellite loci. Based on the results of parentage analysis the larvae were divided into three populations: (1) larvae population with both parents being hatchery-reared fish (=R), (2) larvae population with only a male or a female parent being hatchery-reared fish (=H), and (3) larvae population with no hatchery-reared fish parent (=W). The following analyses were also carried out: (1) assessing the contribution of hatchery-reared offspring to larval resources, and (2) evaluating the genetic effect of stock enhancement on the wild population.

**Results:**

In total, 10.37% and 11.56% of larvae were identified as the offspring produced by hatchery-reared fish released in 2016 and 2017, respectively. In 2017, some of the larvae were assigned unambiguously to hatchery-reared fish released in 2016. In terms of the number of offspring produced, the hatchery-reared fish have shown significant variations. No significant differences were found among all the larvae populations concerning genetic parameters for diversity. High levels of genetic diversity of all larvae populations were obtained. Low *F*_ST_values obtained from pairwise *F*_ST_ analysis, as well as the analysis of molecular variance (AMOVA), revealed high genetic structural similarity among all the larvae populations. The genetic composition of the W larvae population in 2017 was different from that of all other larvae populations (all larvae populations in 2016, and R and H larvae populations in 2017), as demonstrated from the results of STRUCTURE and PCA analyses.

**Conclusion:**

It was demonstrated that hatchery-reared fish are successful in producing the offspring in the natural environment during multiple years, which might assist in increasing the abundance of larvae. The hatchery-reared fish had variations in terms of the success rates on reproduction. Also, the hatchery-reared enhancement program had no significant effect on the genetic diversity or the genetic structure of wild populations. However, the genetic component of the W larvae population in 2017 was changed as compared to 2016, which was not due to the hatchery-reared enhancement program for silver carp. This could be due to flooding, but the specific causes need further studies. Our results clearly show the necessity to continuously inspect the genetic impact of the enhancement program so that historical information can be utilized for further research.

## Introduction

Stock enhancement is becoming increasingly prevalent in both China and other countries as an effective means of fisheries management to deal with the decline of natural resources (Bell et al., 2005; Bell et al., 2006; Guo et al., 2018). Evaluating effectiveness is an integral part of stock enhancement (De Silva & Funge-Smith, 2005). In the stock enhancement program, studies have shown that the stock enhancement activities not only restore the fishery resources but also pose potential risks of reducing the genetic diversity and changing the genetic structure of wild populations in the releasing water areas (Gonzalez et al., 2015; Lorenzen, Beveridge & Mangel, 2012).

Silver carp (*Hypophthalmichthys molitrix*), one of the four major Chinese carps (the other three species are black carp—*Mylopharyngodon piceus*, grass carp—*Ctenopharyngodon idella* and bighead carp—*Hypophthalmichthys nobilis*), is an essential economic fish in China. Their largest native habitat is the Yangtze River which has provided a high commercial catch during the 20th century (Wu, Wang & Cao, 1993). This fish has been over-exploited in recent decades in the middle Yangtze River. Also, the reproductive activity of silver carp has been influenced by the hydrological conditions, such as the water temperature and floods (Duan et al., 2009; Xu et al., 2015). The construction of the Three Gorges Dam has caused a reduction in the severity of the downstream floods (Zhang, 2012), and also a delay in the annual increase of water temperatures. The annual egg abundances of the four major Chinese carps have declined significantly after the construction of the Three Gorges Dam (Duan et al., 2009). A reduction in the numbers of breeding adults may be another reason for the reduction in the egg numbers. To restore these critical natural resources, several measures have been carried out which include establishing a closed fishing season (Chen et al., 2009), the ecological operation of the reservoirs (Xu et al., 2015), and stock enhancement. A hatchery-reared enhancement program for the silver carp has been conducted in the middle Yangtze River since 2010, which releases matured fishes to the river to increase the number of larvae. The hatchery-reared fish are produced by catching the adults from the middle reaches of the Yangtze River as juveniles and rearing them in the National Original Breeding Farm (NOBF). A mature female silver carp could produce hundreds of thousands of eggs, and thus hundreds of matured fishes could supply large numbers of larvae. However, still some problems have not been solved, for example, the confirmation of successful spawning of hatchery-reared adults, the confirmation on the contribution of their offspring to larvae resources in the river, and the genetic effect of the enhancement program on the wild population. All the above information is critical to determining the success of this hatchery-reared enhancement program.

Microsatellites (simple sequence repeat, SSR) are commonly used markers for genetic and genomic studies due to their co-dominance and high levels of polymorphism. To date, they have been successfully used in assessing the release and enhancement of fish, especially in parentage assignment and genetic analyses (Boersen, 2003; Blanco Gonzalez, Nagasawa & Umino, 2008; Kazemi & Finkelstein, 2007). To reduce the time and cost associated with the analyses of SSRs, multiplex polymerase chain reaction (PCR) technique for multiple loci co-amplifying in a single reaction, has been used for multicolor fluorescence genotyping (Henegariu et al., 1997; Oliveira & De, 2002). One such multiplex PCR system for silver carp has already been developed in our laboratory (Li, Cheng & Wang, 2012).

The goals of this study were (1) to assess the contribution of hatchery-reared offspring to larvae resources, and (2) to evaluate the genetic effect of the stock enhancement program on the wild population.

## Material and Methods

### Sampling

**Figure 1:**
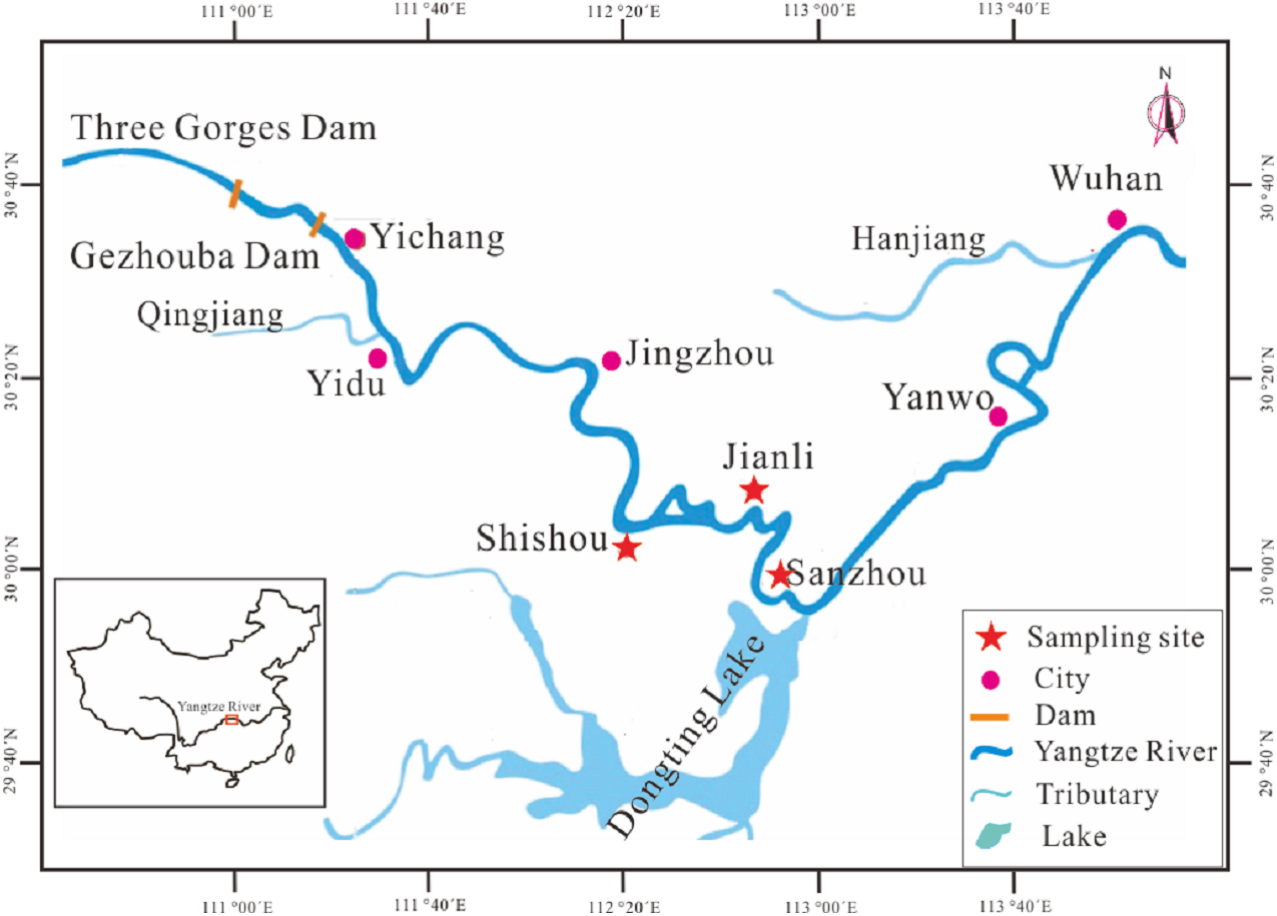
Map showing the sampling locations and other main sites in stock enhancement program in the middle reaches of Yangtze River. Sampling locations: Jianli, Shishou and Sanzhou. Site for catching juveniles of silver carp: Sanzhou and Yanwo. Site for National Original Breeding Farm (NOBF): Jianli and Shishou. Site for releasing: Sanzhou. Site for catching larvae: Sanzhou. Site for spawning ground of silver carp: Yidu.

Juveniles of silver carp were captured from Yanwo and Sanzhou sections in the middle Yangtze River and then reared for 3–5 years in the National Original Breeding Farm (NOBF) located in Jianli (29°35′N, 113°1′E) and Shishou (29°50′N, 113°12′E) Counties, Hubei Province. The matured individuals were selected as hatchery-reared fish and released in Sanzhou section (29°33′N, 112°57′E) of the middle Yangtze River (Fig. 1). The release activity was chosen in late April (closed fishing season) which is one month before the spawning season and aims to reduce the risk of being captured and allows enough time to adapt to natural conditions so that the hatchery-reared fish could spawn in the river. A total of 702 and 827 hatchery-reared fish were released in 2016 and 2017, respectively. The parameters of sex, body length (BL) and body weight (BW) (Table 1) were recorded before release. Meanwhile, the little fin of each fish was clipped and stored in a solution of absolute alcohol for further DNA tests.

**Table 1:** The relevant information recorded for the hatchery-reared populations and confirmed breeders populations of silver carp.

	hatchery-reared				confirmed breeders			
	sex	*N*	BL (cm) (mean ± sd)	BW (kg) (mean ± sd)	sex	*N*	BL (cm) (mean ± sd)	BW (kg) (mean ± sd)
2016	Female	386	65.25 ± 6.36	8.29 ± 2.13	Female	36	64.27 ± 6.29	8.01 ± 1.88
	Male	316	65.37 ± 5.89	8.11 ± 1.96	Male	23	66.26 ± 6.31	8.49 ± 2.21
	Total	702	65.30 ± 6.15	8.21 ± 2.03	Total	59	65.25 ± 6.58	8.32 ± 2.05
								
2017	Female	436	65.43 ± 6.37	8.53 ± 2.12	Female	23	63.48 ± 6.65	8.11 ± 1.88
	Male	391	64.77 ± 5.49	8.18 ± 1.73	Male	14	61.79 ± 5.23	8.43 ± 1.87
	Total	827	65.12 ± 5.97	8.37 ± 1.96	Total	37	62.83 ± 6.13	8.22 ± 1.58

**Notes.**

N number BL body length BW body weight

The annual spawning season for silver carp in the Yangtze River is from May to June (Duan et al., 2009). In the spawning season, most of the matured silver carp migrated to spawning ground in Yidu section (Fig. 1) for reproduction and the fertilized eggs hatch with the flowing water. A station was set up at the releasing site to capture the larvae with a ring net and a trap net as described earlier (Duan et al., 2009). A total of 376 and 493 larvae were collected in 2016 and 2017, respectively, and the collected larvae were preserved in a solution of 95% ethanol for the extraction of DNA and subsequent analysis.

### DNA extraction and microsatellite genotyping

Total genomic DNA was extracted from the alcohol-preserved fins and larvae using a salt-extraction method (Martínez et al., 1998). All specimens were investigated with thirteen microsatellite loci: BL145, BL55, BL109, BL18, BL66, Hym133, Hym230, Hym267, Hym159, BL65, BL106, Hym364, and Hym284 (Table 2), following multiplex PCR protocols as described previously (Li, Cheng & Wang, 2012). Amplification products were genotyped using an ABI 3500XL Genetic Analyzer (Applied Biosystems, Foster City, CA, USA), and the alleles were obtained with GENEMAPPER v. 4.1 (Applied Biosystems).

**Table 2:** Details of thirteen microsatellite loci used in this study (Li, Cheng & Wang, 2012).

LOCUS	Primer sequence (5′–3′)	Size range (bp)	Dye label	Ta (°C)
MI				
BL145	F: GTGATTGGACGGGATGAACTA R: TCTTTCTTTTCTGTCCGAGGG	94∼114	F:5′FAM	60
BL55	F: AAGGAAAGTTGGCTGCTC R: GGCTCTGAGGGAGATACCAC	204∼254	F:5′FAM	
BL18	F: CGAGACAAATAAGGTTGGATA R:CACAAAGAAACTGGAACAAAGAG	110∼164	F:5′TAMRA	
BL109	F: GTGTCCTGGATTCTAGCCG R:CATGAGAGAAACACCTGAACA	212∼262	F:5′HEX	
MII				
Hym230	F: TTGGTGATAAGACGGAAGTG R: ACATTGATAGGCTGGTGGA	274∼377	F:5′HEX	54
Hym267	F: CTATCTGAGAATGCTGCTGTA R: GGGTTAGGCACTTAGTTGTT	218∼252	F:5′TAMRA	
Hym133	F: TCGGATTTACACCACAACTA R: CACTCCCTCAGATTACATTTC	153∼178	F:5′HEX	
BL66	F: TTTGTTTCCGCCGTGGTG R: GGTTCAGGGTTCAATGTCC	311∼341	F:5′FAM	
MIII				
BL65	F: TTAGAGCCATTAGAGGAAAA R: ACACGGAAGCCATTGTTG	301∼353	F:5′FAM	52
Hym159	F: GGCTGCCTGTGAATAA R: CAAGAAGTTGAGGGAGAC	203∼255	F:5′FAM	
BL106	F:TTTAATTCTTCTAGCTGGACACG R: CACTCCTCTTCCCTCGTAAAT	211∼239	F:5′HEX	
Hym364	F: TGGGCTCTAAAGGAAAACAC R: GCTCAAAAGATGCTCCAATAC	363∼397	F:5′HEX	
Hym284	F: ATTCCACTCTGCTTAGGT R: TTGCCGTTACATCCACAC	292∼398	F:5′TAMRA	

### Parentage assignment

Cervus version 3.0 (Marshall et al., 1998) was used to calculate the number of observed alleles (k), observed heterozygosity (Ho), expected heterozygosity (He), polymorphic information content (PIC) and the null allele frequency. Genotyping error rate was set at 1% (as default), and the typing errors were set as 5% in the parentage assignment procedures, which help to reduce the impacts of mutations and null alleles on the analysis of parent-larvae relationships (Marshall et al., 1998). It was more efficient using the single-parent mode than the pair-parent mode in Cervus v 3.0 (Li et al., 2013). According to the recorded gender information (Supplemental Information 1), the parentage analysis was conducted at first by the mode of maternity, followed by the mode of paternity.

The number of potential parents could influence the precision of the assignment to one correct parent. Also, in this study, the number of hatchery-reared fish in 2016 and 2017 were all less than 900. The parentage assignment simulation was carried out using the software in both the modes of paternity and maternity, and the number of candidate parents was set to 900. Ten thousand cycles of simulated assignments were carried out using 95% confidence intervals. After analysis, the assignment rate was still kept at 100% with 900 candidate parents.

In this study, the river-reared potential parents were not sampled and thus if a larva was not assigned to at least one parent, then it could be assumed that non-hatchery-reared fish produced it. Paternity exclusion is a commonly used method for parentage assignment, which is based on Mendelian segregation of alleles, and it allows moderate mismatching and genotyping errors between a progeny and its parental alleles (Vandeputte & Haffray, 2014). For our purpose, larvae with one pair of loci mismatching (LOD > 1) were also considered to be the offspring of hatchery-reared fish.

### Population genetic analysis

To explore the fine genetic diversity and genetic structure, each year the larvae were divided into three populations based on the results of parentage analysis: the R larvae population having larvae with both parents being hatchery-reared fish; the H larvae population with only a male or a female parent being hatchery-reared fish; and the W larvae population for the remaining larvae. The hatchery-reared fish population was also divided into two: confirmed breeders population having hatchery-reared fish with the detection of larvae, and unconfirmed breeders population with the remaining larvae. There were three larvae and one confirmed breeder population in 2016 and 2017 respectively, and overall eight populations were studied.

Box plots were drawn by Origin 6.0 (And, 2010) software to display the distribution of the length and weight of the bodies of two hatchery-reared fish populations. *T*-test (George & Mallery, 2003) was used to evaluate the significant differences between two hatchery-reared fish populations for body length and weight, with the same sex.

Genetic diversity was characterized by the number of alleles (A), expected heterozygosity (He) and observed heterozygosity (Ho), which were calculated by using PopGene32 (version 1.32) (Yeh et al., 1997) software. The allelic richness (Ar) was calculated to correct the variations in the sample sizes by FSTAT ver 2.9 (Goudet, 2002). Regression analysis (George & Mallery, 2003) was used to determine significant differences. Departures from Hardy-Weinberg equilibrium (HWE) at each locus and pairwise fixation index (*F*_ST_) were performed using the Arlequin v 3.0 package (Excoffier, Laval & Schneider, 2005). The analysis of molecular variance (AMOVA) was also performed using Arlequin v 3.0 package. Bonferroni adjustments to the P-values of *F*_ST_ were applied whenever multiple tests were performed. Among populations, the possibility of their structure was examined by clustering techniques based on Bayesian method using STRUCTURE v2.2 (Pritchard, Stephens & Donnelly, 2000). For each value of *K* (*K* = 2–7), ten runs were implemented with a burn of 20,000 in length, followed by 100,000 Markov chain Monte Carlo iterations. Δ*K* was calculated with the obtained standard deviation, which was used to identify the appropriate number of clusters (Evanno, Regnaut & Goudet, 2005). The optimal *K* values were selected by STRUCTURE HARVESTER (http://taylor0.biology.ucla.edu/structureHarvester/). Besides, Principal Component Analysis (PCA) was performed by PCAGEN v 1.2.1 (http://www2.unil.ch/popgen/softwares/pcagen.htm), to examine the patterns of genetic differentiation in the populations.

## Results

### Parentage assignment

**Figure 2:**
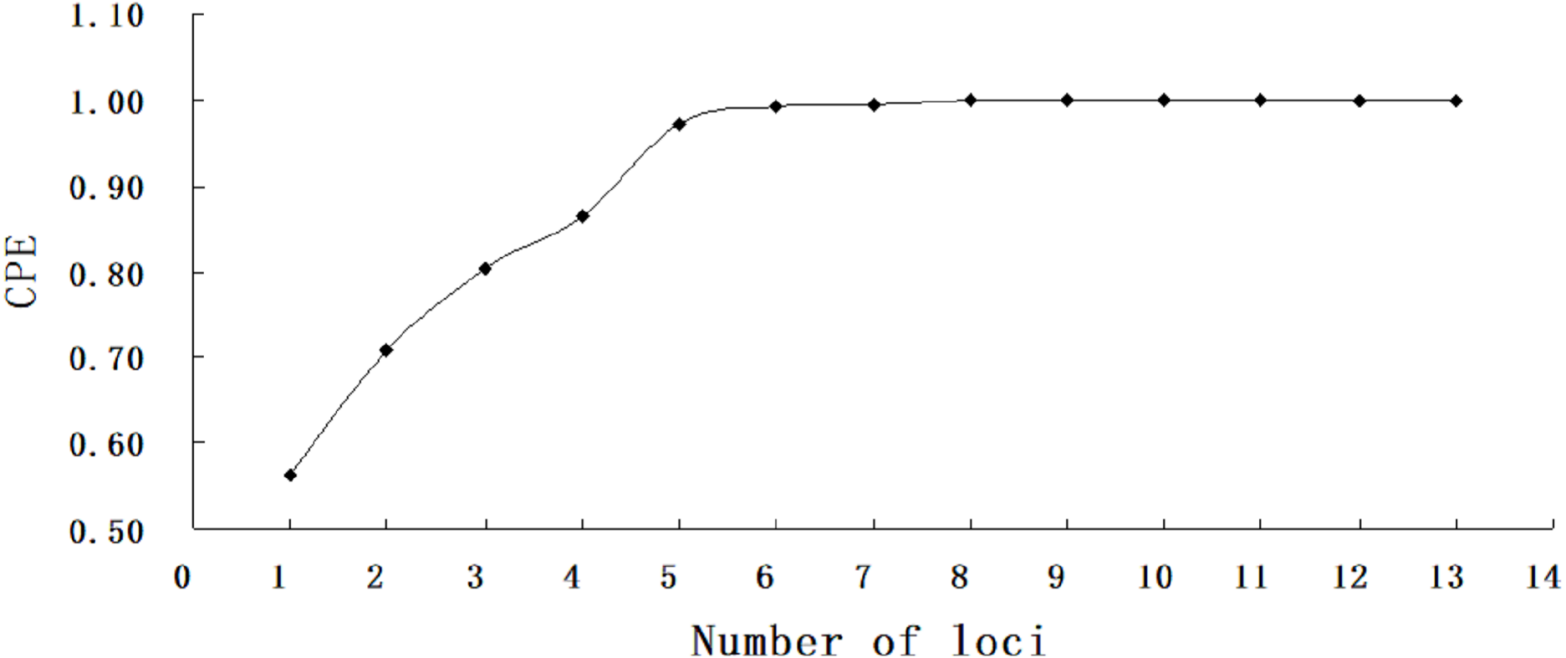
Combined probability of exclusion increasing with number of loci.

A total of 1,529 hatchery-reared fish and 869 larvae were genotyped at thirteen microsatellite loci. The number of allele (k) per locus ranged from 12 in BL145 to 31 in Hym284 (Table 3). The average observed heterozygosity (Ho) and expected heterozygosity (He) were 0.849 and 0.852, respectively. All markers used in the study were highly informative with a high average PIC value (0.835). The absolute values of null allele frequencies were between 0.3% and 4.8%. The combined exclusion power (CPE) achieved 99% correctness and 100% assignment with as few as six loci for no available information from any parents (Fig. 2).

**Table 3:** Summary of characteristics of the thirteen microsatellite loci used in this study.

	k	Ho	He	PIC	F(Null)
BL145	12	0.893	0.815	0.791	−0.048
BL55	25	0.906	0.912	0.905	0.003
BL109	22	0.946	0.921	0.916	−0.014
BL18	26	0.883	0.932	0.928	0.027
BL66	17	0.744	0.698	0.649	−0.042
Hym133	16	0.731	0.753	0.72	0.008
Hym230	27	0.87	0.892	0.882	0.011
Hym267	21	0.754	0.73	0.699	−0.025
Hym159	18	0.786	0.827	0.806	0.021
BL65	28	0.877	0.919	0.913	0.021
BL106	18	0.902	0.861	0.846	−0.024
Hym364	14	0.86	0.887	0.876	0.014
Hym284	31	0.88	0.926	0.921	0.024
Average	21.2	0.849	0.852	0.835	

**Notes.**

K number of observed alleles Ho observed heterozygosity He excepted heterozygosity PIC polymorphic information content F(Null) estimated null allele frequency

The results of parentage analysis showed that 39 larvae in 2016 could be unambiguously assigned to hatchery-reared fish, which included six R larvae and 33 H larvae. Fifty seven larvae in 2017 could be unambiguously assigned to hatchery-reared fish, which included one R larva with two breeders in 2016, one R larva with two breeders in 2017, four R larvae with one breeder each in 2016 and 2017, 19 H larvae with one breeder in 2016, and 32 H larvae with one breeder in 2017 (Table 4). The sum of R and H larvae accounted for 10.37% and 11.56% of the larvae in 2016 and 2017, respectively. In this study, the remaining larvae were produced by non-hatchery-reared fish.

**Table 4:** Parentage assignment analysis results for the larvae in 2016 and 2017.

		larvae		
Matching to		2016	2017	
*n*		376	493	
confirmed breeders (Br) year		2016	2016	2017
R	Br 2016 × Br2016 or Br 2017 × Br2017	6	1	1
	Br 2016 × Br2017	–	4	
H		33	19	32
W		337	436	
Br (Female)		23	15	23
Br (Male)		19	6	14
Br (Total)		59		37

### Differential parental contributions of hatchery-reared fish to larvae and their growth performance

**Figure 3:**
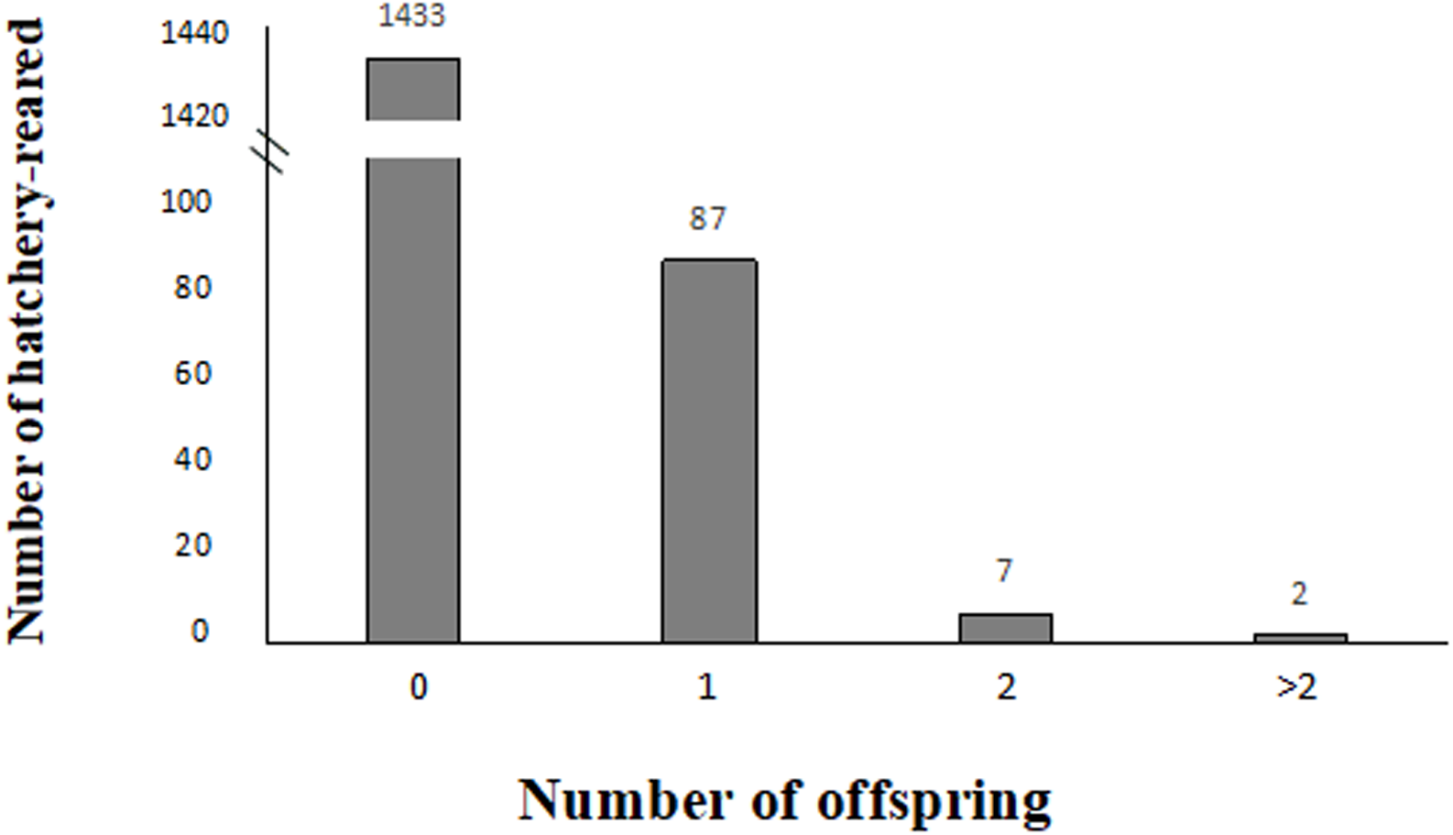
Variation in reproductive success of hatchery-reared based on the parentage assignment results.

In 2016, 63 larvae were produced from 59 confirmed breeders, which consisted of 36 females and 23 males, whereas in 2017, 37 larvae were produced from 37 confirmed breeders, which consisted of 23 females and 14 males, as shown in Table 4. In this study, among all the studied hatchery-reared fish, 1,433 unconfirmed breeders did not contribute to larvae (93.72% which included 763 females and 670 males), and 87 confirmed breeders contributed to one larva (5.68% which included 54 females and 33 males), and 7 confirmed breeders contributed to two larvae (0.45% which included four females and three males), and two confirmed breeders contributed to over two larvae (0.13% which included 1 female and 1 male) (Fig. 3).

**Figure 4:**
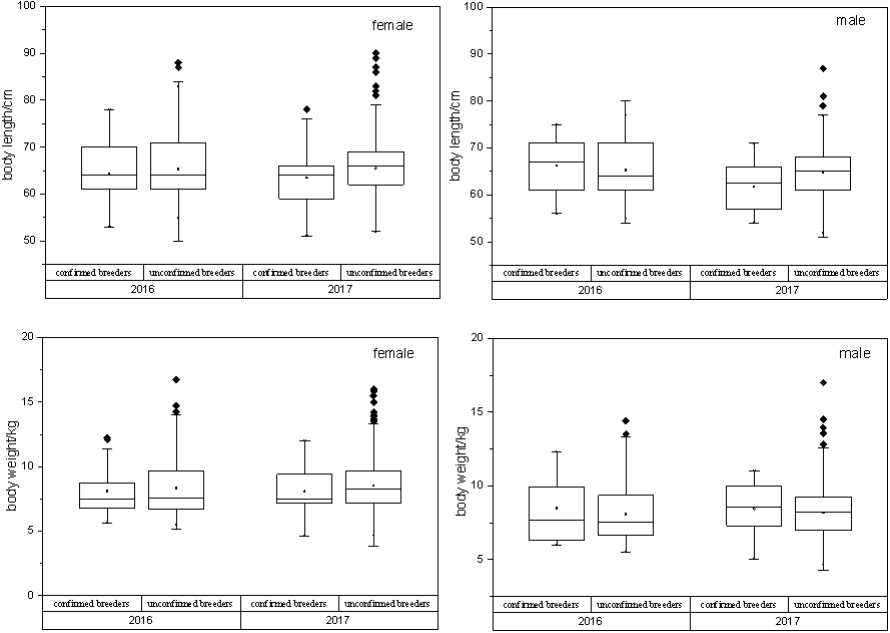
Relationship between confirmed breeders populations and unconfirmed breeders populations in terms of body length/ weight, sex and release year. *T*-test results showed that no statistically significant differences between two breeders groups were observed, with the same sex (P_*BL*−*M*_ = 0.11, P_*BL*−*F*_ = 0.61, P_*BW*−*M*_ = 0.22, P _*BW*−*F*_ = 0.28). BL, body length; BW, body weight; F, female; M, male.

In case of all confirmed breeders, the observed variations in the sex, body length and body weight are shown in Table 1. Box plots were used to display the distribution of body length and body weight of all the confirmed and unconfirmed breeders’ populations (Fig. 4). *T*-test results showed statistically no significant difference (*p* > 0.05) between two populations with the same sex.

**Table 5:** Genetic diversity of confirmed breeders (Br), R larvae, H larvae and W larvae at 13 microsaellite loci. Confirmed breeders (Br) were hatchery-reared having larvae detected. R larvae were larvae that both parents were the hatchery-reared. H larvae were larvae with only a male or a female parent was the hatchery-reared. W larvae were larvae the rest.

		Confirmed Breeders (Br)		R larvae		H larvae		W larvae	
		Br2016	Br2017	R2016	R2017	H2016	H2017	W2016	W2017
BL145	A	7	8	5	6	8	8	11	11
	Ar	4.6	5.1	5.0	6.0	4.9	5.0	5.0	5.2
	Ho	0.929	1.000	1.000	1.000	0.879	0.882	0.884	0.743
	He	0.779	0.802	0.803	0.818	0.782	0.772	0.777	0.784
BL55	A	16	17	6	6	16	16	22	19
	Ar	7.3	7.8	6.0	6.0	7.5	7.2	8.2	7.4
	Ho	1.000	0.966	1.000	1.000	0.970	0.824	0.958	0.828
	He	0.890	0.913	0.879	0.864	0.899	0.881	0.923	0.897
BL109	A	16	17	8	8	15	16	21	21
	Ar	7.5	7.6	8.0	8.0	8.1	7.6	8.0	8.2
	Ho	1.000	0.983	1.000	0.833	0.939	0.824	0.961	0.876
	He	0.903	0.903	0.924	0.924	0.923	0.899	0.915	0.923
BL18	A	16	21	7	7	19	21	26	26
	Ar	7.9	8.0	7.0	7.0	7.5	8.3	8.5	8.6
	Ho	0.929	0.914	1.000	1.000	0.909	0.882	0.841	0.828
	He	0.909	0.914	0.879	0.909	0.888	0.923	0.926	0.933
BL66	A	5	5	3	4	4	5	9	17
	Ar	3.4	3.2	3.0	4.0	3.1	3.5	3.5	5.1
	Ho	0.905	0.793	1.000	0.667	0.849	0.628	0.819	0.598
	He	0.645	0.608	0.667	0.561	0.614	0.630	0.645	0.776
Hym133	A	8	7	3	3	5	9	11	16
	Ar	4.5	4.0	3.0	3.0	4.1	4.5	4.4	5.8
	Ho	0.810	0.810	1.000	0.833	0.909	0.745	0.839	0.686
	He	0.749	0.671	0.682	0.621	0.702	0.711	0.729	0.802
Hym230	A	15	12	7	8	12	14	17	26
	Ar	6.7	6.4	7.0	8.0	6.6	6.7	6.7	7.8
	Ho	0.929	0.931	1.000	1.000	0.970	0.824	0.937	0.774
	He	0.873	0.861	0.894	0.939	0.872	0.868	0.872	0.911
Hym267	A	7	9	6	2	10	11	14	20
	Ar	4.2	4.1	6.0	2.0	4.9	3.9	5.7	5.5
	Ho	0.762	0.759	1.000	0.333	0.879	0.667	0.832	0.708
	He	0.659	0.656	0.818	0.303	0.728	0.630	0.810	0.774
Hym159	A	9	8	4	5	9	13	15	16
	Ar	4.3	4.8	4.0	5.0	5.3	5.2	5.1	6.0
	Ho	0.952	0.931	1.000	0.667	0.909	0.784	0.851	0.600
	He	0.746	0.775	0.742	0.803	0.760	0.783	0.768	0.844
BL65	A	15	15	8	6	15	18	22	27
	Ar	7.2	7.1	8.0	6.0	7.8	7.7	7.7	8.7
	Ho	0.929	0.931	1.000	0.833	1.000	0.882	0.942	0.767
	He	0.891	0.890	0.909	0.758	0.914	0.905	0.909	0.936
BL106	A	11	10	5	6	9	10	12	13
	Ar	5.9	5.6	5.0	6.0	6.1	5.9	6.0	5.8
	Ho	0.952	1.000	1.000	1.000	1.000	0.843	0.917	0.853
	He	0.848	0.819	0.849	0.879	0.859	0.843	0.845	0.838
Hym364	A	10	10	7	6	9	12	12	14
	Ar	6.2	6.2	7.0	6.0	6.3	6.2	6.5	6.7
	Ho	0.857	0.948	1.000	1.000	1.000	0.902	0.892	0.811
	He	0.862	0.861	0.894	0.849	0.866	0.849	0.872	0.876
Hym284	A	16	17	7	8	14	17	24	26
	Ar	7.7	7.8	7.0	8.0	7.5	7.9	8.0	8.6
	Ho	0.952	0.948	0.833	1.000	0.939	0.824	0.944	0.716
	He	0.907	0.913	0.909	0.939	0.905	0.911	0.916	0.933
mean	A	11.6	12.0	5.8	5.8	11.2	13.1	16.6	19.4
	Ar	6.0	6.0	5.8	5.8	6.1	6.1	6.4	6.9
	Ho	0.916	0.916	0.987	0.859	0.935	0.808	0.894	0.753
	He	0.820	0.814	0.835	0.782	0.824	0.816	0.839	0.863

**Notes.**

A number of alleles per locus Ar allelic richness Ho observed heterozygosity He expected heterozygosity

### Genetic variation and differentiation among confirmed breeders and larvae populations

The obtained results based on eight populations were analyzed at 13 microsatellite loci (Table 5). W larvae populations had the highest number of alleles (A) per locus, while R larvae populations had the lowest number of alleles per locus. The discrepancy between them was statistically significant (*p* < 0.05, Supplemental Information 2). The values of average allelic richness (Ar) supported the difference in the sample size among populations which varied between 5.8 in two R populations to 6.9 in W2017. The mean expected heterozygosity (He) values were similar in all eight populations, ranging from 0.782 in R2017 to 0.863 in W2017. No significant difference was found among eight populations in terms of Ar and Ho (*p* > 0.05). All the average values of observed heterozygosity (Ho) revealed high levels of genetic variation, and the R larvae population had the highest number, followed by H larvae population and then by the W larvae population per year.

Low *F*_ST_ values were observed among the samples of silver carp, suggesting that there was high homogeneity among all populations (Table 6). W2016 had the highest *F*_ST_ value which was significantly different from R2017 (*F*_ST_ = 0.03, *P* = 0.00). The differences were also significant between W larvae populations and most of the other populations. In particular, W2017 had low but significantly different *F*_ST_ values from other populations except for R2016. The results of AMOVA analysis showed that 97.98% of the genetic variations occurred within populations while only 2.02% of the genetic variation occurred across eight populations, and a significant but low genetic differentiation was found (*F*_ST_ = 0.020 and *P* < 0.001, Table 7).

**Table 6:** Pairwise *F*_ST_ values among confirmed breeders (Br2016 and Br2017), R larvae (R2016 and R2017), H larvae (H2016 and H2017) and W larvae (W2016 and W2017).

	Br2016	Br2017	R2016	R2017	H2016	H2017	W2016	Br	R	H	2017
Br2017	0.006^*^										
R2016	−0.012	0.000									
R2017	0.012^*^	−0.002	0.012								
H2016	0.001	0.008^*^	−0.021	0.017^*^							
H2017	0.007^*^	−0.002	0.002	−0.005	0.012^*^						
W2016	0.008^*^	0.015^*^	−0.012	0.03^*^	−0.001	0.019^*^					
W2017	0.025^*^	0.017^*^	0.014	0.019	0.022^*^	0.013^*^	0.025^*^				
R								−0.005			
H								−0.001	−0.001		
W								0.009^**^	0.004	0.005^**^	
2016											0.021^***^

**Notes.**

Br, Br2016+Br2017; R, R2016+R2017; H, H2016+H2017; W, W2016+W2017; 2016, Br2016+R2016+H2016+W2016; 2017, Br2017+R2017+H2017+W2017.

* *P* < 0.002 after Bonferroni correction, *k* = 28 ** *P* < 0.008 after Bonferroni correction, *k* = 6 *** *P* < 0.05

**Table 7:** Analysis of molecular variance (AMOVA) for silver carp populations estimated from SSR markers using ARLEQUIN v 3.0.

Groups	Source of variation	Variance	% of variance	Fixation index	*P*-value
One group including 8 populations	Among populations	0.101	2.02		
	Within populations *F*_ST_	4.935	97.98	0.020	<0.001
2016 vs. Br2017, R2017, H2017 vs. W2017	Among groups *F*_CT_	0.110	2.19	0.021	0.002
	Among populations within groups *F*_SC_	0.002	0.06	0.001	0.015
	Within populations *F*_ST_	4.935	97.76	0.022	<0.001

**Figure 5:**
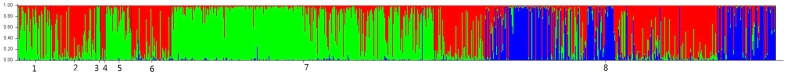
Result of the STRUCTURE analysis based on Bayesian clustering of eight silver carp populations. Here, we show result of *K* = 3. The number 1–8 represents the following populations: Br2016, Br2017, R2016, R2017, H2016, H2017, W2016 and W2017.

The STRUCTURE analysis showed the number of genetic clusters, and the obtained results revealed that the highest Δ*K* value was obtained for *K* = 3 (Fig. 5), with four populations in 2016 forming one cluster and fish from populations in 2017 assigned among other two clusters. A visual inspection of the STRUCTURE results showed that W2017 and other populations in 2017 did not cluster together and this is confirmed from the proportion of eight populations in each of the three inferred clusters (Table 8).

**Table 8:** Proportion of eight populations of each of the three inferred clusters.

Population	Inferred cluster			Number of individuals
	1	2	3	
Br2016	0.328	0.662	0.009	42
Br2017	0.702	0.287	0.010	58
R2016	0.148	0.846	0.006	6
R2017	0.904	0.080	0.017	6
H2016	0.185	0.806	0.009	33
H2017	0.778	0.196	0.026	51
W2016	0.151	0.833	0.016	337
W2017	0.499	0.131	0.370	436

**Figure 6:**
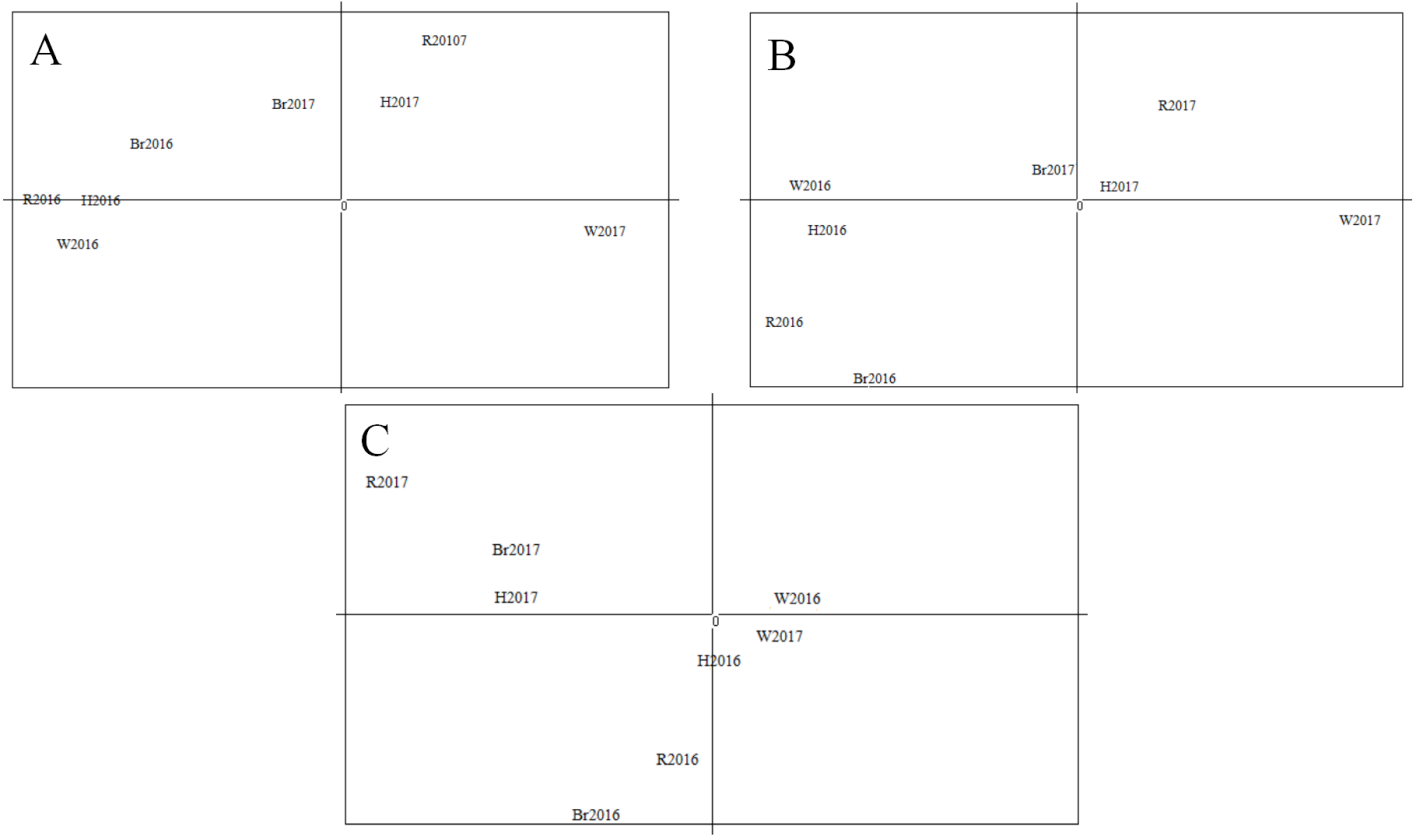
Results of PCA with allele frequencies. The plots display the three principal components accounting for 68.76% (PC1), 17.59% (PC2) and 4.8% (PC3) of the total variation: (A) PC1(*X*-axis) and PC2 (*Y*-axis); (B) PC1 (*X*-axis) and PC3 (*Y*-axis); (C) PC2(*X*-axis) and PC3 (*Y*-axis).

The Principal Component Analysis (PCA) results are shown in Fig. 6. The first component (68.76% of the overall variation) and the second component (17.59% of the overall variation) analysis separated four populations in 2016, W2017 and the rest of the sample populations (Fig. 6A). Meanwhile, the first and third components (4.8% of the overall variation) analysis demonstrated a similar result (Fig. 6B). The results obtained from the second and third components’ analyses (Fig. 6C) were not considered as the proportion of their overall variation was low (<25%). Hence, the analysis based on three main components disclosed three different groups. It could be seen that the results from PCA were in good agreement with that of STRUCTURE. Moreover, these groupings were supported by the results of AMOVA analysis as shown in Table 7 (*F*_CT_ = 0.021 and *P* = 0.002, *F*_SC_ = 0.001 and *P* = 0.015).

## Discussion

As a powerful and effective genetic marker, microsatellites are useful for parentage assignment and population genetics in many species (Novel et al., 2010; Jeong et al., 2010; Wang et al., 2008; Borrell et al., 2014). The thirteen microsatellite loci used in this study had high heterozygosity and PIC values (>0.8), low, null alleles frequency (<5%) and high CPE (>99.9%). They provided statistical evidence for the parentage approach to enhance the silver carp.

In this study, we chose sexually matured hatchery-reared fish as stock and released them to spawn in natural waters. R larvae confirmed that the hatchery-reared fish could produce larvae and increase the breeding population directly, while H larvae indicated that the hatchery-reared fish could find the right spawning ground and mate with wild individuals randomly. Some larvae in 2017 were unambiguously assigned to hatchery-reared fish that were released in 2016, which confirmed that the hatchery-reared fish could spawn and successfully produce larvae for multiple years under appropriate conditions. Fishing effort, migration, and mortality are the common factors affecting the dynamics of the stocks and may explain the reason for the offspring produced by hatchery-reared fish made up of 10.4% and 11.6% of the total larvae in 2016 and 2017, respectively (Blanco Gonzalez, Nagasawa & Umino, 2008). The W larvae dominated in the river, which reveals that it is hard for hatchery-reared fish with low proportions to replace wild fish in natural waters.

Generally, when calculating the recapture rate, the target of catching is mainly related to the fish released before (Hvidsten & Hansen, 2010; Soivio & Virtanen, 1985). In this study, the recapture rates of larvae were calculated due to the release of sexually matured fish with high fecundity. The data obtained were similar to the report for black sea bream (*Acanthopagrus schlegelii*) released at Daio Bay each year over three years (2000–2002) (Blanco Gonzalez, Nagasawa & Umino, 2008). However, the rate was much higher than that of other species released in China such as Japanese croaker (*Nibea japonica*) (0.11%∼1.1%) (Liang et al., 2010), great yellow croaker (Pseudosciaena crocea) (0.1%∼6.45%) (Ma et al., 2016), and swimming crabs (*Portunus trituberculatus*) (6.43%∼7.54%) (Xie et al., 2014). Many factors have been reported to have an impact on the recapture rates such as the marking method, recapture position, water environment and releasing size (Xie et al., 2014). The *T*-test results from this study showed that the body length/weight had no significant effect on the recapture rate of hatchery-reared fish. This shows that other factors should also be taken into consideration in the design of future recapturing experiments.

Frequently, breeders with variations in their reproductive success have been found (Borrell et al., 2014; Marine et al., 2008; Novel et al., 2010). Hierarchical crosses conducted between six females and 30 males of European seabass in artificial fertilization found that some breeders account for certain progeny, while other breeders had no contribution (Novel et al., 2010). The results of parentage assignment from this study indicated a similar situation in the wild environment. Silver carp produce drifting eggs, and the water environment of different sections had discrepancies. The passive drift-diffusion process of larvae was influenced by the discharges of the river and the morphology of channel, and the characteristics of drift were entirely different in different sections of the river (Araujo-Lima & Oliveira, 2010; Li et al., 2011). The preference might occur at the time of fertilization or during the stages of larvae. It could be related to different reasons such as competition of sperm, quality of gamete, different hydrological conditions and survival environments (Luo et al., 2016; Miller et al., 2014).

Sustaining genetic diversity within the populations is an essential goal in the stock enhancement programs for further adaptation to a changing environment (Allendorf & Luikart, 2007). Long-term monitoring of the possibility of genetic drift and other genetic changes are necessary for stock enhancement programs. So far, several results have been reported on the genetic effects of stock enhancement with different types of molecular markers (Shan et al., 2017; Sanchezlamadrid, 2004; Romo et al., 2006). Some studies have shown that hatchery fish may have characteristics such as lower genetic diversity and reduced frequency of private alleles, compared with wild fish (Araki et al., 2007; Kong & Li, 2007). In this study, wild-origin fish were released to avoid the adverse effects of releasing hatchery-origin fish. The obtained molecular genetic parameters (A, Ar, He and Ho) showed a high level of genetic diversity of all larvae populations and were higher than the previously demonstrated (Wang et al., 2008; Zhu et al., 2007). Since variations in the number of samples were large, there were significant differences among some of the populations in A (*p* < 0.05). No significant differences appeared in Ar and Ho (*p* > 0.05), although there was a significant difference among some of the populations in He (*p* < 0.05). Similar to the research results of black sea bream (*Acanthopagrus schlegeli* i) stocks in Japan (Blanco Gonzalez, Nagasawa & Umino, 2008), a high genetic similarity was found among all the larvae populations of silver carp in terms of genetic diversity.

Consistent with the results of earlier studies (Wang et al., 2008; Zhu et al., 2007), the results of pairwise *F*_ST_ analysis and AMOVA analysis revealed a significant but a low genetic difference in all the populations. However, the results of both STRUCTURE and PCA analyses indicated that the genetic component of W2017 was differentiated from other populations. The results of the Bayesian clustering analysis performed with STRUCTURE showed that each population consisted of three inferred clusters, and the proportion of the third inferred cluster (the blue signature in the STRUCTURE plot) in W2017 was higher than that of other populations. The possibility of such a condition caused by our enhancement program is small due to the proportion of the third inferred cluster in the confirmed breeder population is very low. It has been found that the floods occurred in the middle reaches of the Yangtze River in 2016, and reservoirs near the release of Yichang flood waters caused a large number of cultured fish to enter the Yangtze River (Chen et al., 2018). In this case, the genetic diversity of the silver carp population in Yichang decreased. Therefore, it is speculated that the wild gene pool of silver carp in the sample site had been disturbed by a large amount of escaped fish or other released fish, especially the escape of hatchery fish caused by the catastrophic flood in 2016 (Zhu et al., 2009; Gao et al., 2017). The third inferred cluster in W2017 represents larvae that were likely produced from the parents that came from a closed hatchery program. However, to obtain a better understanding, specific reasons need to be further studied in the future.

## Conclusion

This study is the first attempt in describing the effectiveness of hatchery-reared enhancement of silver carp and was conducted in the middle Yangtze River using thirteen microsatellite loci. It was demonstrated that the hatchery-reared fish were successful in producing larvae in the natural environment in multiple years after being released, and perhaps assisting in increasing the abundance. Variations in the reproductive success of hatchery-reared fish were found. The hatchery-reared enhancement program had no significant effect on the genetic diversity and genetic structure of the wild population. However, the genetic components of W larvae population in 2017 changed from 2016 for other reasons. It is obligatory to continuously monitor the genetic impact of the enhancement program to provide more information for further research.

##  Supplemental Information

10.7717/peerj.6836/supp-1 Supplemental Information 1 Biological parameters of confirmed breeders and hatchery-reared

10.7717/peerj.6836/supp-2 Supplemental Information 2 T test results of Parameters with significant differences

10.7717/peerj.6836/supp-3 Supplemental Information 3 SSR data of confirmed breeders and larvae produced by them

## Additional Information and Declarations

### Data Availability

The following information was supplied regarding data availability: The raw measurements are available in Files S1 and S3.
