# Supplementary material for: Hatchery-reared enhancement program for silver carp (Hypophthalmichthys molitrix) in the middle Yangtze River: monitoring the effectiveness based on parentage analysis
Source: PeerJ. 2019 May 6;7:e6836. doi: 10.7717/peerj.6836 (PMC6507896; doi:10.7717/peerj.6836)
Supplement: Supplemental Information 2 [file peerj-07-6836-s002.docx]

**Supplementary material.**

**T test results of Parameters with significant differences.**

|  | P values | |
| --- | --- | --- |
|  | Na | Ho |
| Br2016-Br2017 | 1.000 | 1.000 |
| Br2016-R2016 | 0.086 | 0.354 |
| Br2016-R2017 | 0.084 | 1.000 |
| Br2016-H2016 | 1.000 | 1.000 |
| Br2016-H2017 | 1.000 | 0.286 |
| Br2016-W2016 | 1.000 | 1.000 |
| Br2016-W2017 | 0.198 | 0.019 |
| Br2017-R2016 | 0.043 | 0.441 |
| Br2017-R2017 | 0.042 | 1.000 |
| Br2017-H2016 | 1.000 | 1.000 |
| Br2017-H2017 | 1.000 | 0.227 |
| Br2017-W2016 | 1.000 | 1.000 |
| Br2017-W2017 | 0.360 | 0.014 |
| R2016-R2017 | 1.000 | 0.348 |
| R2016-H2016 | 0.166 | 1.000 |
| R2016-H2017 | 0.007 | 0.000 |
| R2016-W2016 | 0.000 | 0.048 |
| R2016-W2017 | 0.000 | 0.000 |
| R2017-H2016 | 0.163 | 1.000 |
| R2017-H2017 | 0.007 | 0.291 |
| R2017-W2016 | 0.000 | 1.000 |
| R2017-W2017 | 0.000 | 0.019 |
| H2016-H2017 | 1.000 | 0.049 |
| H2016-W2016 | 1.000 | 1.000 |
| H2016-W2017 | 0.104 | 0.002 |
| H2017-W2016 | 1.000 | 1.000 |
| H2017-W2017 | 1.000 | 1.000 |
| W2016-W2017 | 1.000 | 0.162 |
